# Brain magnetic resonance imaging findings among children with epilepsy in two urban hospital settings, Kampala-Uganda: a descriptive study

**DOI:** 10.1186/s12880-022-00901-7

**Published:** 2022-10-06

**Authors:** Denise Apolot, Geoffrey Erem, Rita Nassanga, Daniel Kiggundu, Crescent Max Tumusiime, Anneth Teu, Alex Mwesigwa Mugisha, Robert Sebunya

**Affiliations:** 1 Department of Radiology, School of Medicine, Makerere University College of Health Sciences, Kampala, Uganda; 2 Clinical Epidemiology Unit, College of Health Sciences, Makerere University, Kampala, Uganda; 3 Department of Pediatrics, Mother Kevin Postgraduate Medical School, Uganda Martyrs University School of Medicine, Kampala, Uganda; 4 Department of Radiology, Mother Kevin Postgraduate Medical School, Uganda Martyrs University School of Medicine, Kampala, Uganda; 5 St.Francis hospital, Nsambya, Uganda

**Keywords:** Epilepsy, Magnetic resonance imaging, Structural abnormality, Acquired, Congenital

## Abstract

**Background:**

Epilepsy is one of the most common neurological conditions in children worldwide. Its presentation is heterogeneous, with diverse underlying aetiology, clinical presentation, and prognosis. Structural brain abnormalities are among the recognized causes of epilepsy. Brain Magnetic Resonance Imaging (MRI) is the imaging modality of choice for epilepsy workup.

We aimed to determine the prevalence and describe the structural abnormalities identified in the brain MRI studies performed on children with epilepsy from two urban hospitals in Kampala, Uganda.

**Methods:**

This was a cross-sectional descriptive study performed at two urban hospital MRI centres. The study population was 147 children aged 1 day to 17 years with confirmed epilepsy. Brain MRI was performed for each child and a questionnaire was used to collect clinical data.

**Results:**

The prevalence of structural abnormalities among children with epilepsy was 74.15% (109 out of 147). Of these, 68.81% were male, and the rest were female. Among these, the majority, 40.14% (59 of 144) were aged 1 month to 4 years. Acquired structural brain abnormalities were the commonest at 69.22% with hippocampal sclerosis (HS) leading while disorders of cortical development were the most common congenital causes.

An abnormal electroencephalogram (EEG) was significant for brain MRI abnormalities among children with epilepsy with 95% of participants with an abnormal EEG study having epileptogenic structural abnormalities detected in their brain MRI studies.

**Conclusion and recommendation:**

Two-thirds of children with epilepsy had structural brain abnormalities. Abnormal activity in the EEG study was found to positively correlate with abnormal brain MRI findings. As such, EEG study should be considered where possible before MRI studies as a determinant for children with epilepsy who will be having imaging studies done in the Ugandan setting.

## Background

Epilepsy is an enduring condition emanating from an imbalance between excessive neuronal excitation and deficient inhibition leading to a predisposition to recurrent seizures [1]. This common neurological disorder affects 70 million people globally, with low to lower-middle income (L&LMICs) countries bearing the lion’s share [2, 3]. Over half of those affected by this condition are children [2, 3].

The International League Against Epilepsy (ILAE) recognises six etiological categories among which is structural brain abnormalities [4].

MRI is considered the investigation of choice in epilepsy work up, particularly for structural brain abnormalities, with the use of high-quality MRI, special protocols, sequences and gadolinium administration [5]. In Sub-Saharan Africa, significant challenges exist in both etiological diagnosis and management of epilepsy. This could in part be due to the limited access to neuroimaging services facilities [6, 7].

Various structural brain abnormalities are associated with epilepsy. They may be classified as acquired and congenital abnormalities. Acquired abnormalities include Hippocampal Sclerosis (HS) and abnormalities resulting from brain insults i.e. infection, trauma, hypoxia, etc. [8, 9].

Congenital abnormalities include malformations of cortical development (MCD) and low grade cerebral neoplasms (such as cortical tumours) [10]. The MCD result from disruption in the neurodevelopment process which may occur at neuronal proliferation, migration or cortical organisation stages. These disruptions can result in numerous MCD such as hemimegalencephaly, lissencephaly, heterotropia, polymocrogyria and schizencephaly [11].

Cerebral neoplasms are commonly associated with epilepsy in 1–3% of children presenting with new onset seizures [12]. Recognised epileptogenic neoplasms include Dysembryoplastic neuroepithelial tumours (DNET). These constitute about 10% of all tumours associated with intractable epilepsy [13]. Others such as pleomorphic xanthoastrocytoma (PXA) may occur with cortical dysplasia or other cortical tumours particularly gangliogliomas [14]. On MRI scan, these lesions are commonly localised in the cerebral cortex or in the mesial temporal areas [15]. Oligodendrogliomas often are located in the mesial temporal and superficial frontal regions.

Various types of seizures are associated with over 20–45% of cerebral arteriovenous malformations (AVMs)[16].The most common vascular abnormality reported in patients with intractable epilepsy is the cavernous angioma. MRI with susceptibility weighted imaging (SWI) is useful in their diagnosis [17].

Despite the above, there is limited data on the structural brain abnormalities among children with epilepsy in our setting. This presents a challenge to targeted management of this condition.

As such, this study aimed to determine the prevalence and describe the structural brain abnormalities in children with epilepsy, with the goal of improving management through targeted therapies and early causative diagnosis as well as prognostic counselling.

## Materials and methods

This was a cross sectional descriptive study involving children below 18 years with epilepsy who had been referred for brain MR imaging at two urban hospitals in Kampala, Uganda between January and October, 2021.

At the time of this study, Kampala had five Health Units performing MRI studies. Of these MRI units, one was equipped with a 3 T MRI while two were equipped with 1.5 T MRI scan machines and two others with 0.5 T machines. The units with 3 T and 0.5 T machines were not included in the study as this would affect uniformity of the reported images when compared with those from the units with 1.5 T machines. Of the three remaining MRI units, two had specialised paediatric neurology services and one did not. As such, this study was conducted at two of these sites with the specialised neurology clinics namely;Nsambya and Nakasero hospital Radiology departments. Nsambya hospital is a 361-bed capacity private-not-for-profit hospital located in the southern part of Kampala city approximately 3 km from the city center. It offers both outpatient and in-patient services. It has a radiology department equipped with 1.5 T MRI (Siemens AG,Germany), 128 slice CT machine, four ultrasound machines, conventional X-ray machine and mammography machine. The department on average receives 20–40 children with seizures for MRI evaluation per month the majority of whom are under the age of 5 years.

Nakasero Hospital is an 80 bed capacity private-for-profit hospital in Kampala, the capital of Uganda. It is located on AkiiBua Road, Nakasero Hill, Kampala Central Division, North of the City's central business district. Imaging services offered at this center include plain radiography, ultrasonography, Computed Tomography (CT), 1.5 T Magnetic Resonance Imaging (Siemens AG,Germany), Mammography and fluoroscopy services. The department does an average of 5 brain MRI studies per month on children with epilepsy.

## Study procedure

Institutional Approval was granted by the Makerere University School of Medicine Research and Ethics Committee (REC REF NO.2021-006). Hospital approval was also sought from the two Hospital managements. All study activities and procedures were conducted Per the Good Clinical and Laboratory Practice starting from January to October 2021.

Identification of the participants was done by the study team in the reception areas of the MRI units. Patients and caretakers of patients doing brain magnetic resonance imaging studies due to epilepsy were informed about the study and those who consented were screened and consecutively enrolled until the required sample size was obtained. Among study participants aged eight years or older, assent was also sought to participate in the study. The clinical data was collected from the MR imaging request forms by the principal investigator and any missing information was obtained by the research team (PI or the research assistant) by interviewing the caregivers of study participants or the study participants (those who gave assent) using a structured pretested questionnaire. Detailed information with regard to demographic profile, history, clinical examination findings, other imaging findings and other important parameters were recorded in a predesigned data collection sheet.

The study participants were screened for metallic implants, which are contraindicated in MRI studies, and only those without implants had the MRI study done. MRI was performed in a 1.5 T Siemens scanner using an established epilepsy protocol. An anatomic, thin slice volumetric T1-weighted gradient-recalled-echo sequence, axial and coronal T2-weighted sequence, fluid attenuated inversion recovery (FLAIR) sequence (axial), and high resolution oblique coronal T2-weighted imaging of the hippocampus (fast or turbo spin echo weighted sequence) were acquired with a maximum slice thickness not exceeding 4–5 mm. Gadolinium contrast enhanced studies were done in cases where it was indicated. All images were stored digitally and reviewed at a later time. Images were reviewed by the principal investigator and 2 qualified radiologists with approximately 10 years of experience each by viewing images on a console, and any abnormality was reported with final decision reached by consensus. In cases where there was disagreement between the two primary radiologist interpretations, images were reviewed by the PI with a pediatric neurologist and a third senior radiologist with 15 years of experience, who adjudicated a final decision.

In cases EEGs had been done, they were reported by an EEG technician and a paediatric neurologist with training in clinical neurophysiology. Diagnosis and classification of epilepsy was made on electroclinical ground whenever possible. Seizures and epilepsy were classified using International Classification of Epileptic Seizures proposed by ILAE in 2017. All the data was entered on the data collection form.

### Data management

The raw data was securely stored to maintain confidentiality. The forms were crosschecked and edited for errors or missed data. With the help of a statistician, data was entered into a computer using Epi info 7 for storage and analysis. SPSS software version 20 was used for further analysis.

### Data analysis

The prevalence of structural abnormalities identified on brain MRI studies of children with epilepsy were obtained as the proportion of children with structural abnormalities on brain MRI out of the total number of study participants. Whilst the MRI structural abnormalities were described and categorized into acquired and congenital.

Bivariate analysis was used to determine the factors associated with brain structural abnormalities among children with epilepsy.

The dependent variable was presence or absence of structural abnormalities identified on brain MRI studies done among children with epilepsy. The independent variables were social demographic and clinical factors.

## Results

### General clinical data

We recruited a total of one hundred and forty-seven participants with epilepsy following a brain MRI study done at either of the two the study sites. Majority (86%) of our study participants were recruited from Nsambya hospital MRI centre and the rest were from Nakasero hospital.

One hundred and forty-four participants had an MRI study done with designated epilepsy protocols (97.96% of 147). The mean age was 5.82 (SD = 4.29) with the majority of participants in the one to four (1–4) years old age category as shown in Table 3.

### Prevalence of MRI abnormalities

The prevalence of MRI abnormalities among the children with seizures studied was 74.15% (109/147) with a 95% CI 66.38–80.65%. Four out of 147 participants had “incidental findings” of sinusitis and or rhinitis with no structural brain abnormalities and were designated as normal.

### Description of MRI abnormalities

**Fig. 1:**
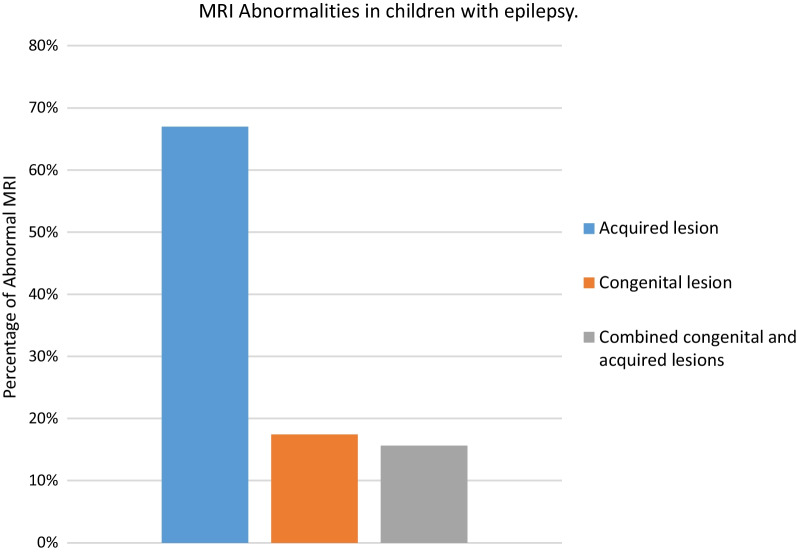
MRI abnormalities classified as congenital and acquired among children with epilepsy whose brain MRI studies were done in two urban hospitals in Kampala, Uganda

**Fig. 2:**
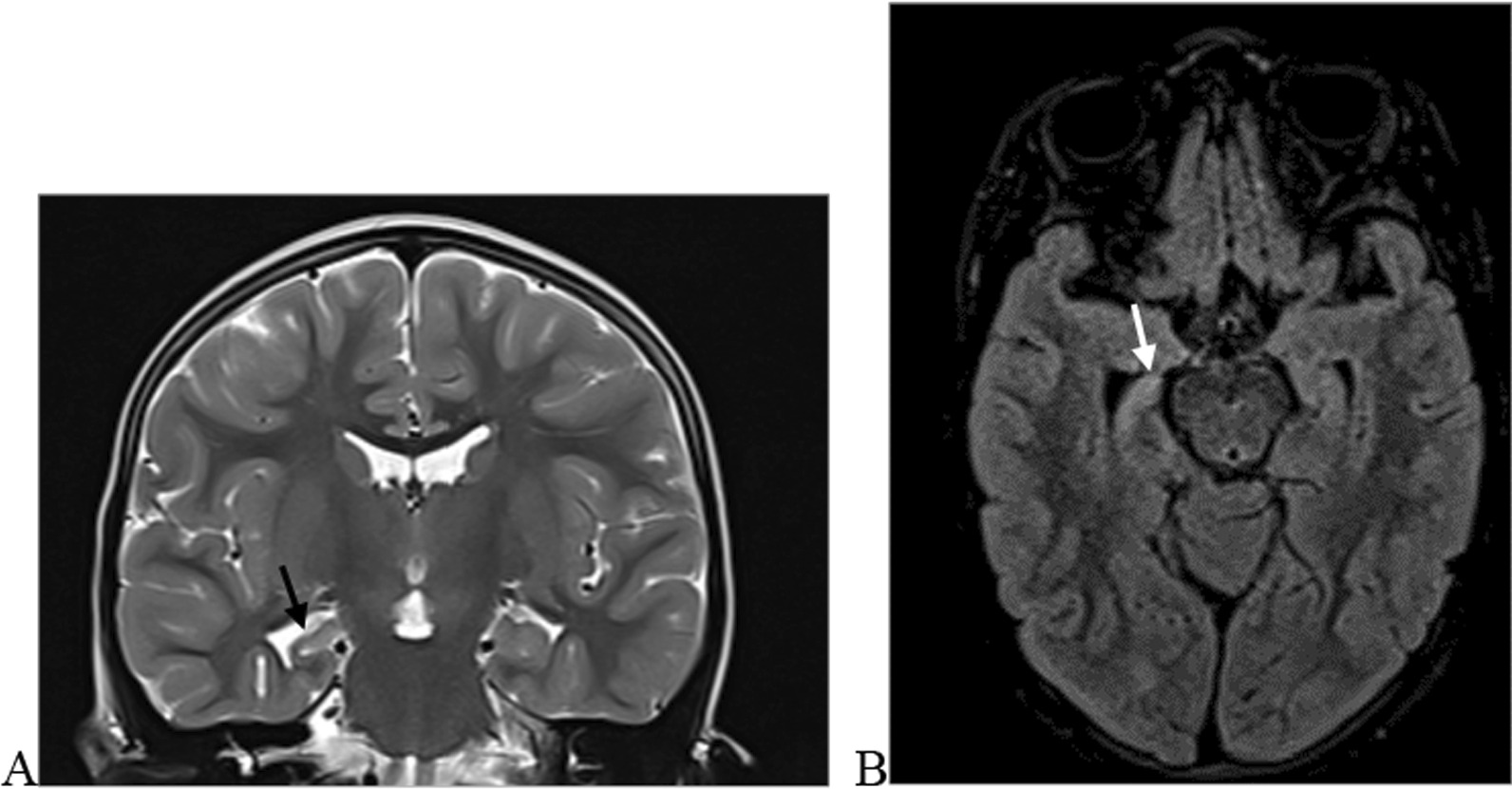
Right hippocampal sclerosis in a four-year-old male with focal seizures. Coronal T2 weighted image (WI) (**A**) and axial T2 FLAIR WI (**B**) images demonstrating an atrophied right hippocampus with dilated ipsilateral choroidal fissure and temporal horn of the lateral ventricle (arrow) and increased signal intensity on FLAIR due to gliosis (arrow) when compared to the contralateral hippocampus

**Fig. 3:**
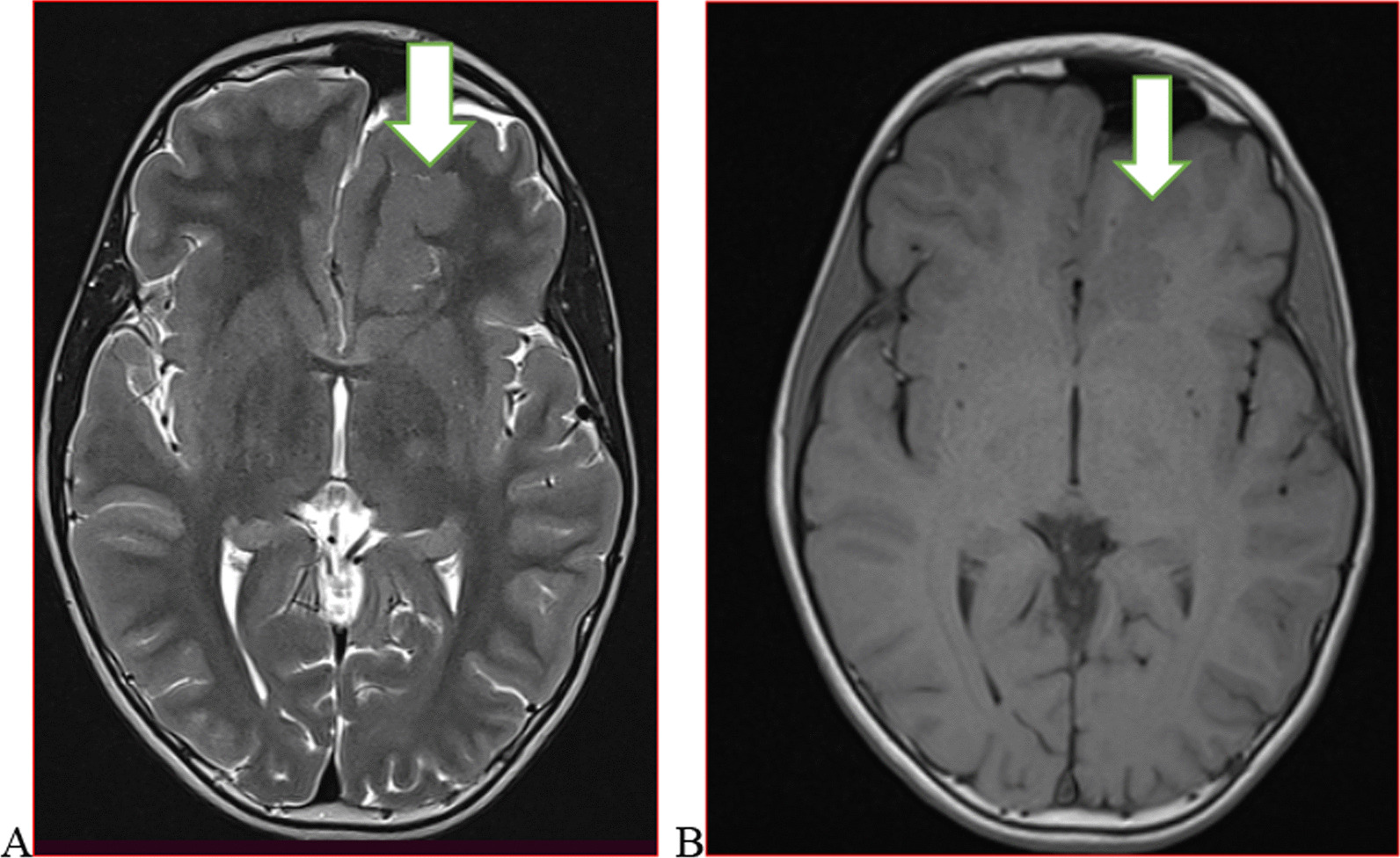
Thirteen-year-old male with focal seizures. Axial T2 (**A**) and T1 (**B**) weighted images demonstrating abnormally thickened left frontal cortex with blurred grey/white matter junction (arrow) consistent with focal cortical dysplasia-a malformation of cortical development

**Fig. 4:**
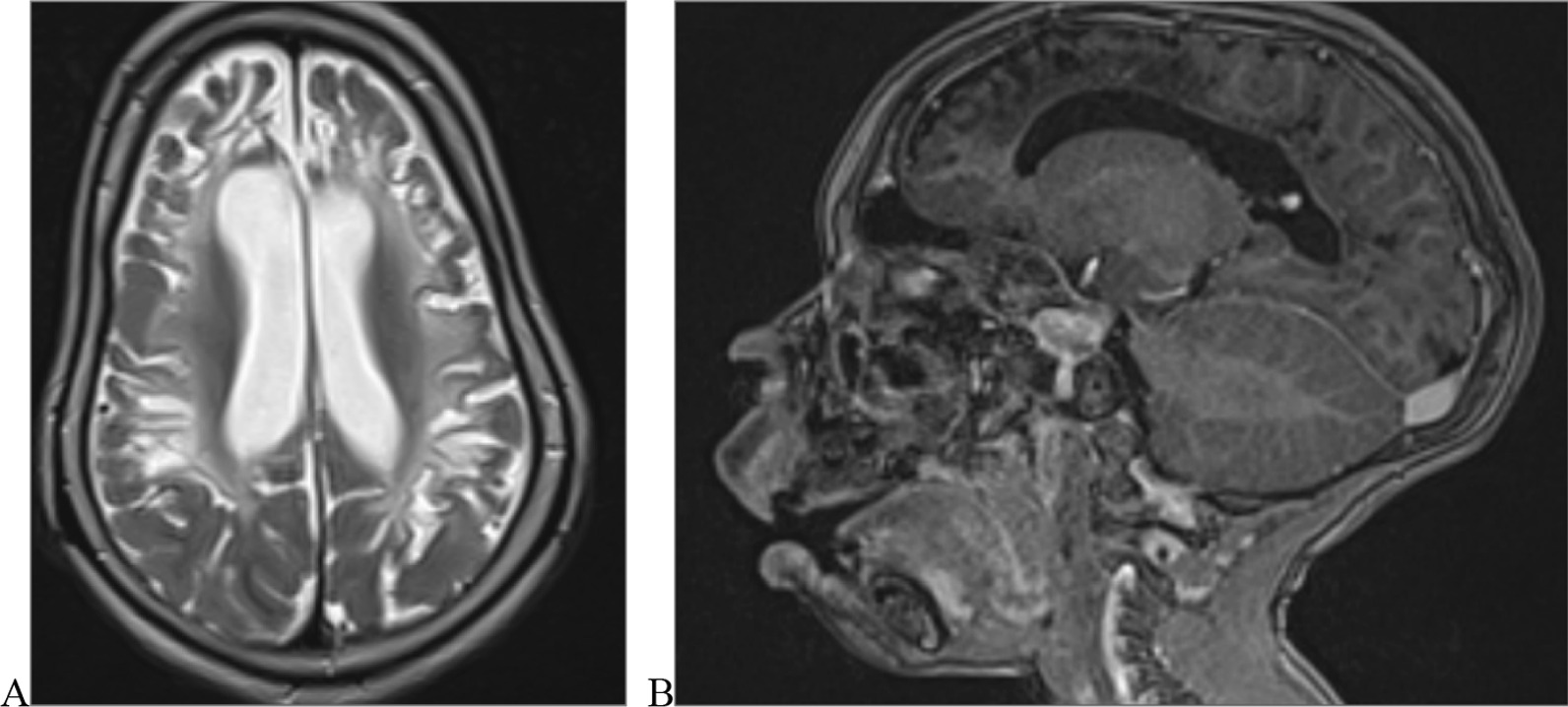
Seven year old male with epilepsy and developmental delay.Axial T2 weighted brain MRI (**A**) and sagittal MPRAGE (**B**) showing bilateral extensive fronto-parietal multicystic encephalomalacia with mushroom shaped gyri(ulegyria) in an infant who was born at term in a case of severe chronic ischaemic insult(hypoxic ischaemic encephalopathy)

**Fig. 5:**
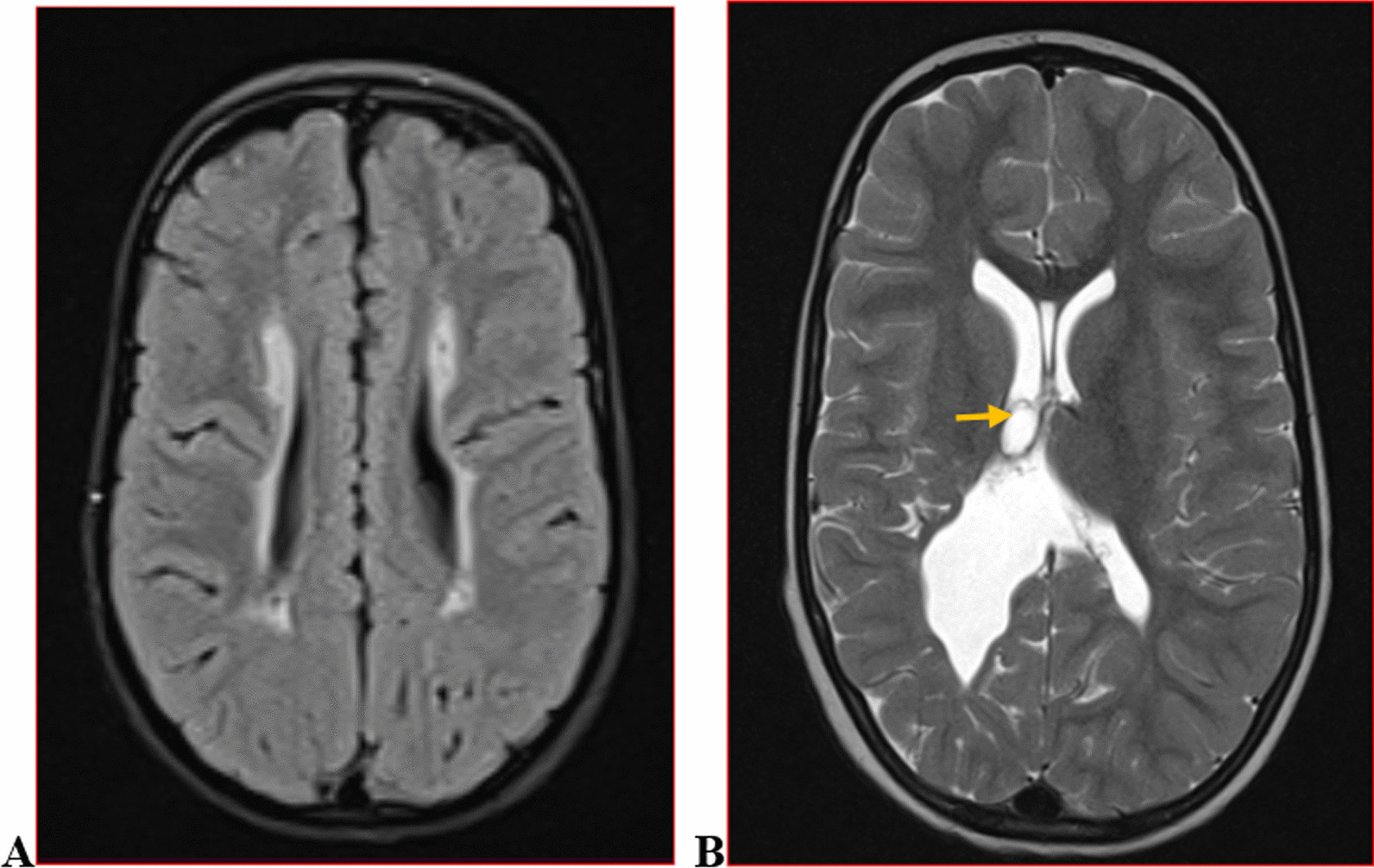
Typical PVL (**A**) with symmetrical FLAIR hyperintense periventricular rim representing periventricular gliosis with associated thinning of the white matter. Atypical PVL (**B**) assymetrical thinning of white matter with apparent dilation and irregular outline of the trigone and the posterior horn of the right lateral ventricle. Note is made of a right intraventricular cyst(arrow) which was an incidental finding

**Fig. 6:**
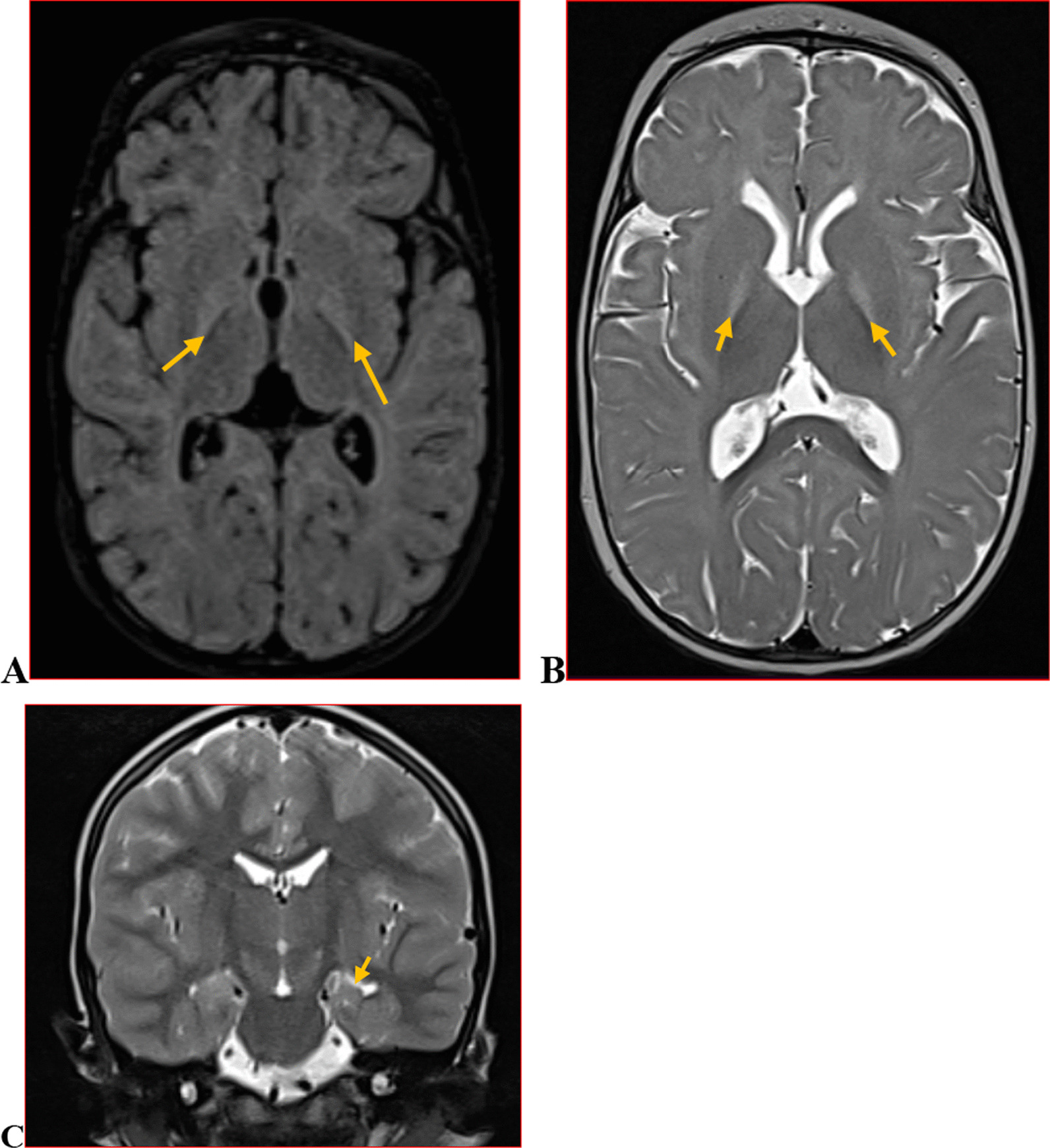
Symmetric globus pallidus T2/FLAIR hyperintensity(arrows) (**A** and **B**) in a 6 year old female with history of infantile jaundice. Findings are in keeping with Kernicterus.Additionally,the patient had left hippocampal atrophy depicted in **C** (arrow)

**Fig. 7:**
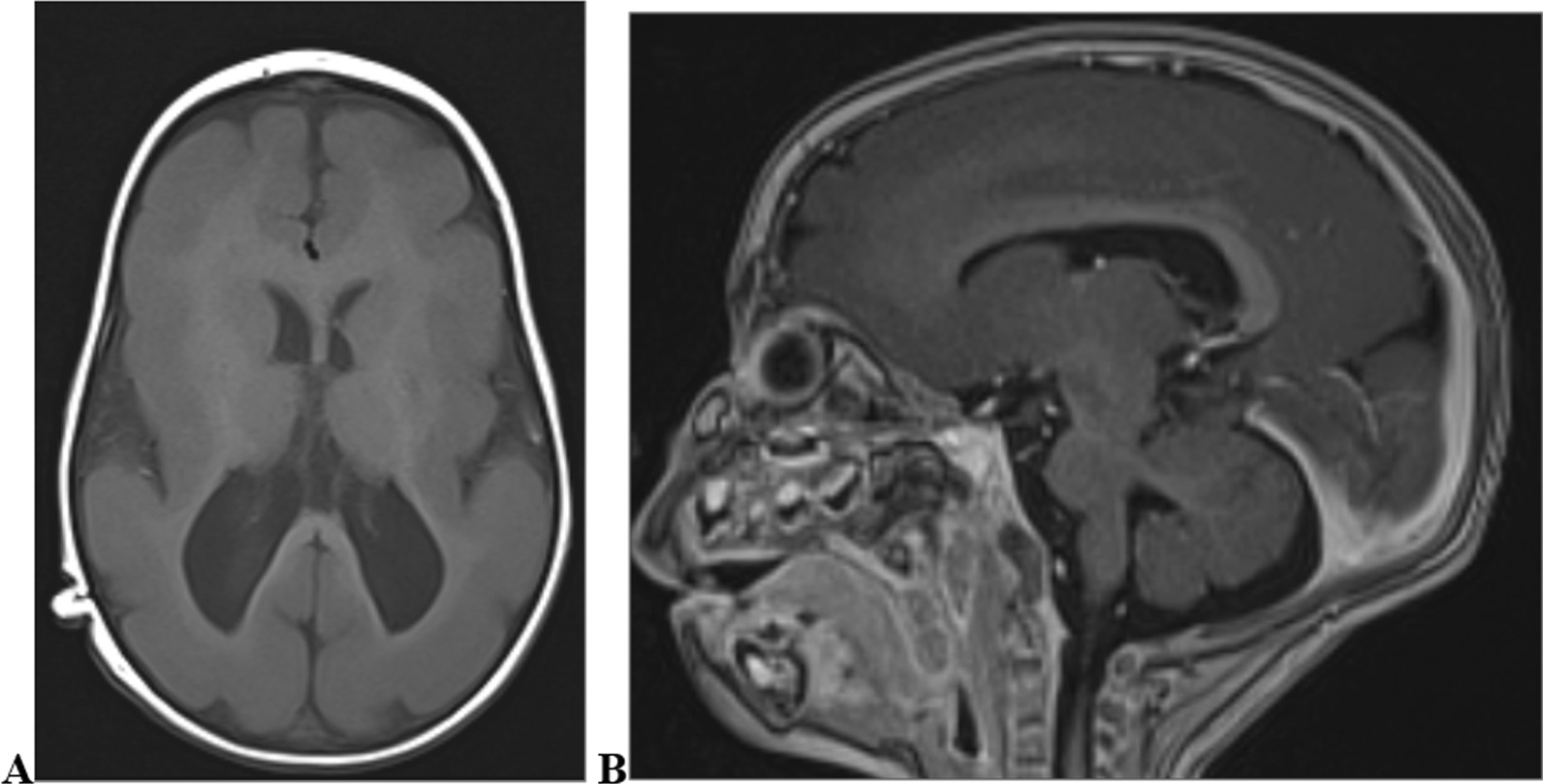
Lissencephaly in a ten-month old male with focal seizures. T1 weighted axial (**A**) and sagittal (**B**) images demonstrating smooth cortical surface with few shallow sulci (pachygyria), uniformly thickened cortex and classic figure-of-8 appearance in axial image due to shallow Sylvian fissures

**Fig. 8:**
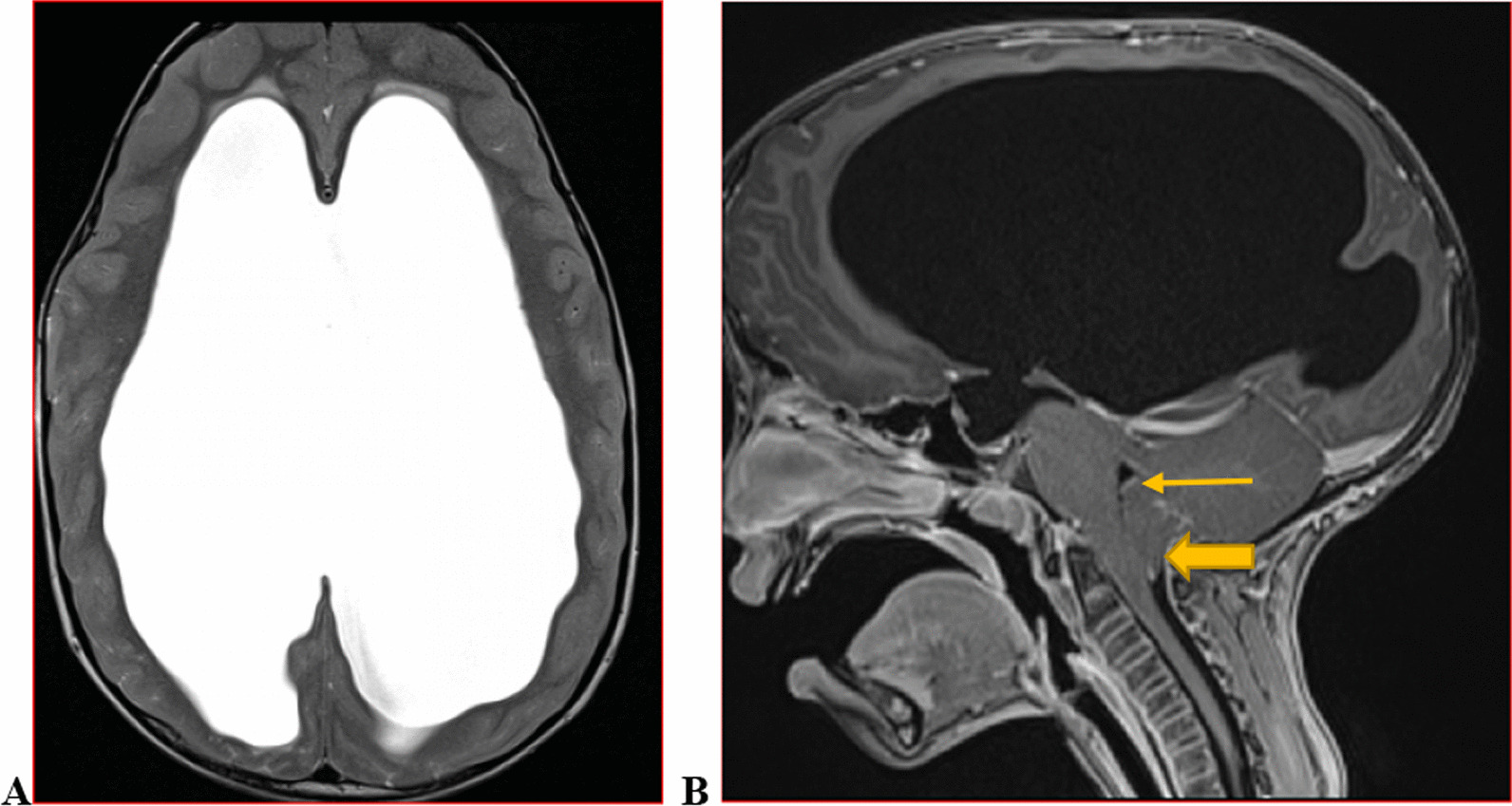
Nine year old male with increased head circumference and epilepsy since early childhood T1 and T2-weighted axial brain MRIs demonstrate grossly dilated lateral ventricles with absent interventricular septum (**A**); 4th ventricle is spared (arrow). Sagittal T1-weighted image (**B**) shows compression of the cerebellum9narrow arrow) with tonsillar herniation(wide arrow). This is a case of hydrocephalus due to aqueductal stenosis with elevated ICP

**Fig. 9:**
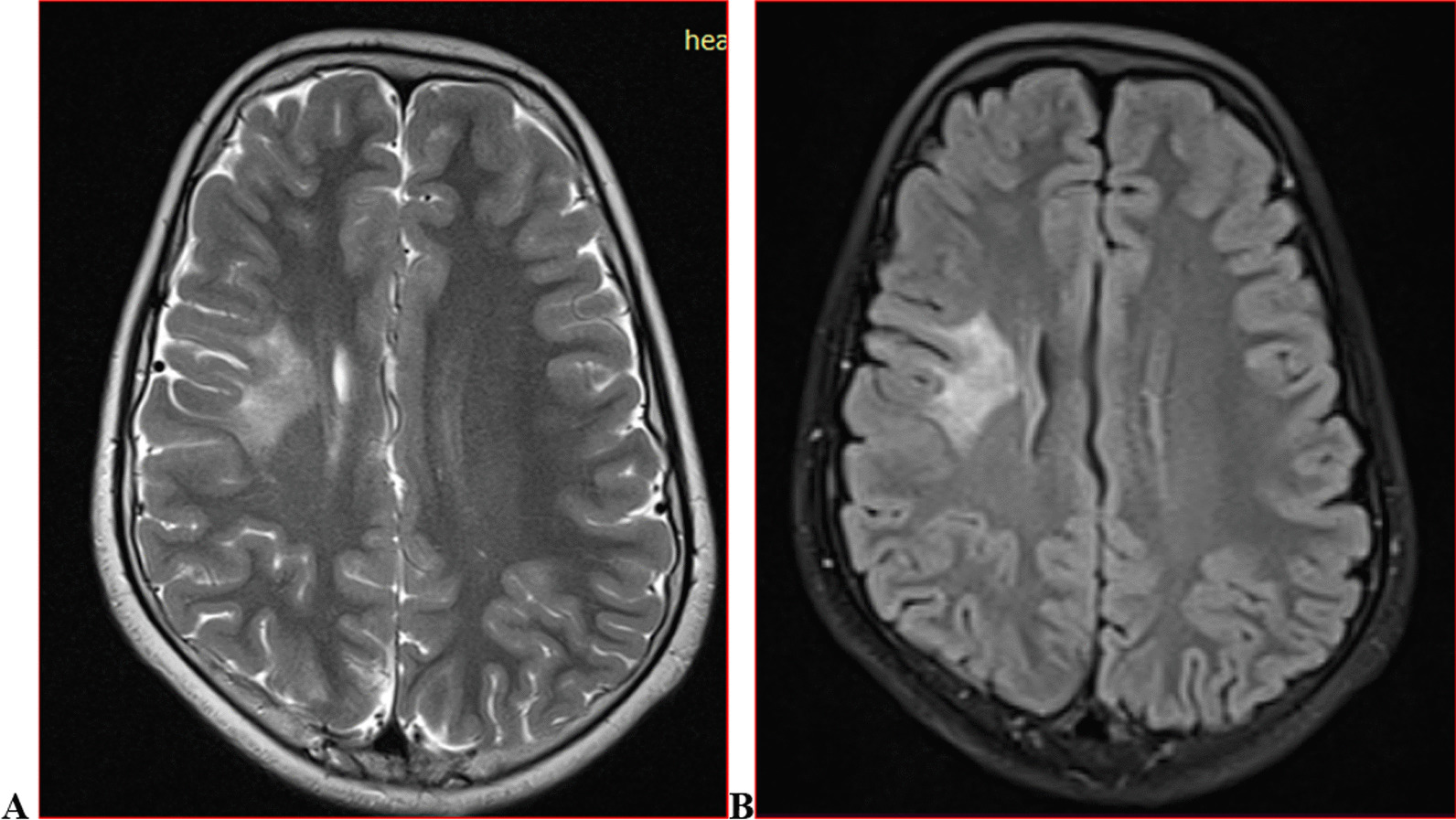
Twelve year old male with epilepsy brain axial MRI (T2/FLAIR) (**A** and **B** respectively) demonstarting subcortical white matter T2/FLAIR hyperintensity with a broad base towards the bottom of sulcus and tapering towards the lateral ventricle. The overlying gyrus appears dysplastic with local widening of the subarachnoid space. These findigs were highly sugestive of Focal Cortical Dysplasia

**Fig. 10:**
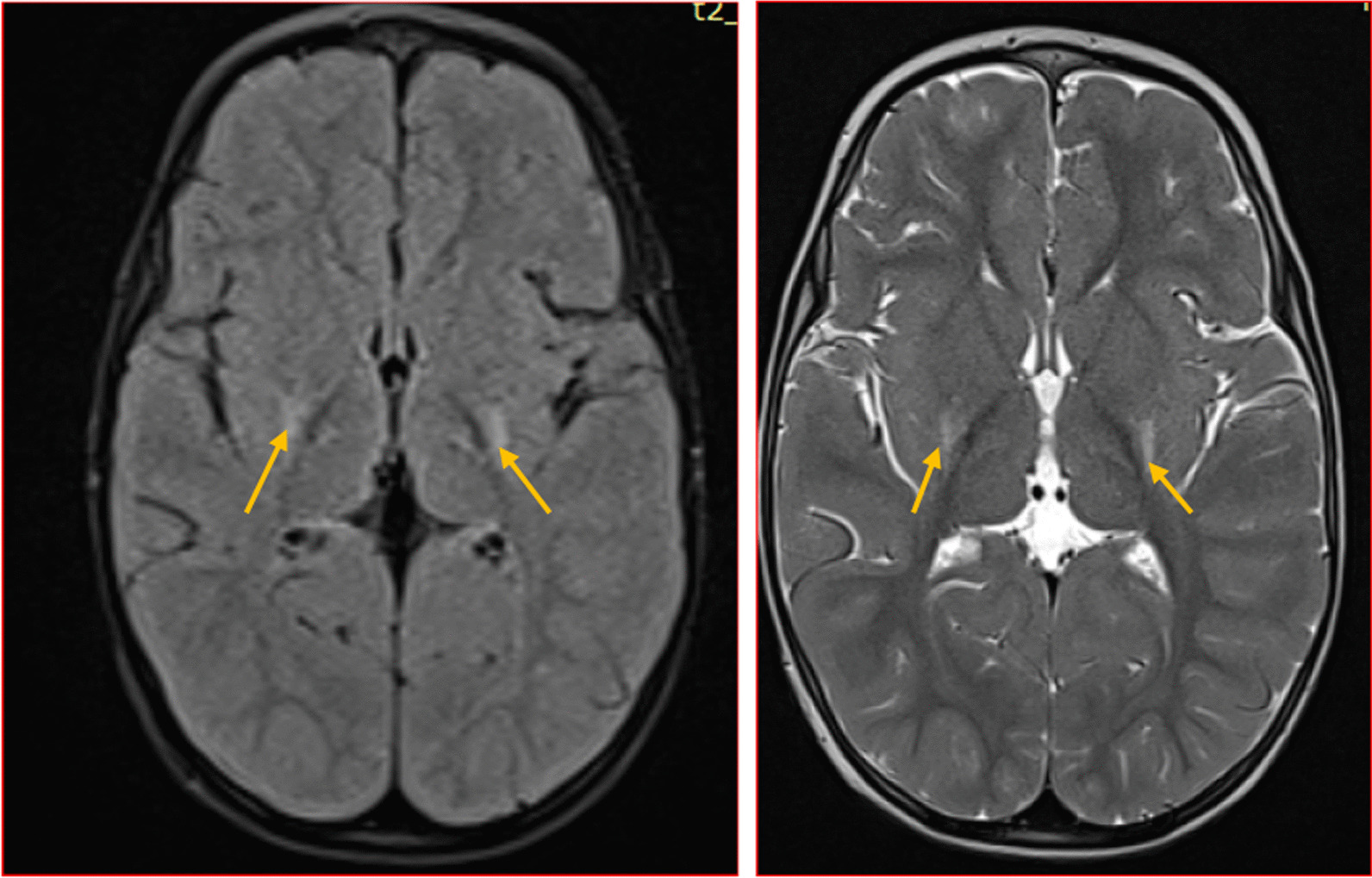
Two year old female with abnormal hand movements. Axial (T2/FLAIR) brain MRI demostrating symmetrical dorsal putaminal T2/FLAIR hyperintensities(arrows) highly suspicious for neuro-metabolic disorder

**Fig. 11:**
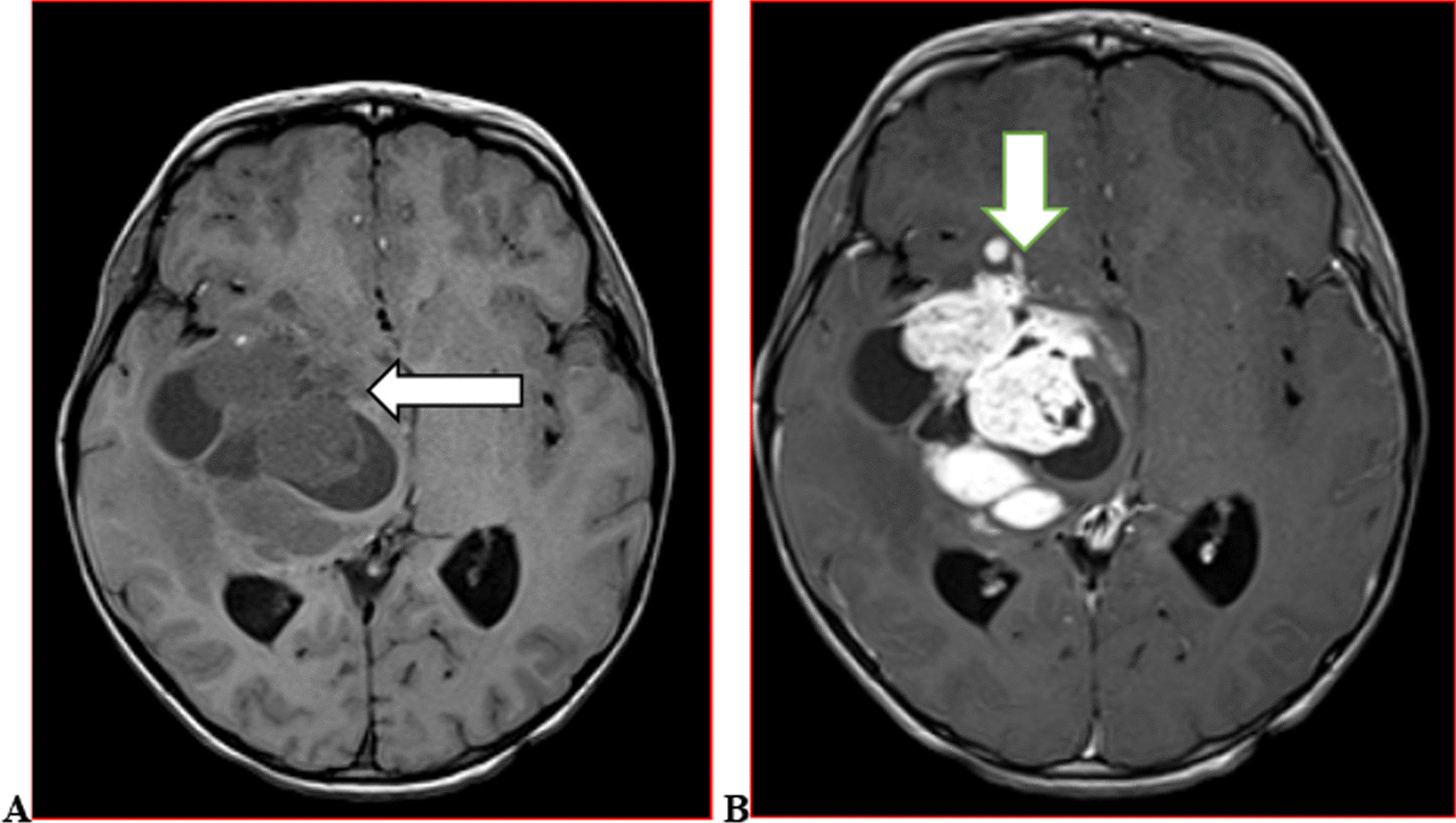
Six year old male with epilepsy. Axial T1 weighted brain MR images pre (**A**) and post (**B**) gadolinium contrast demonstrating a heterogenous mass in the right temporal lobe with extension to ipsilateral basal ganglia and thalamus (arrows) with avid enhancement of the solid component in a case of a tumor

**Fig. 12:**
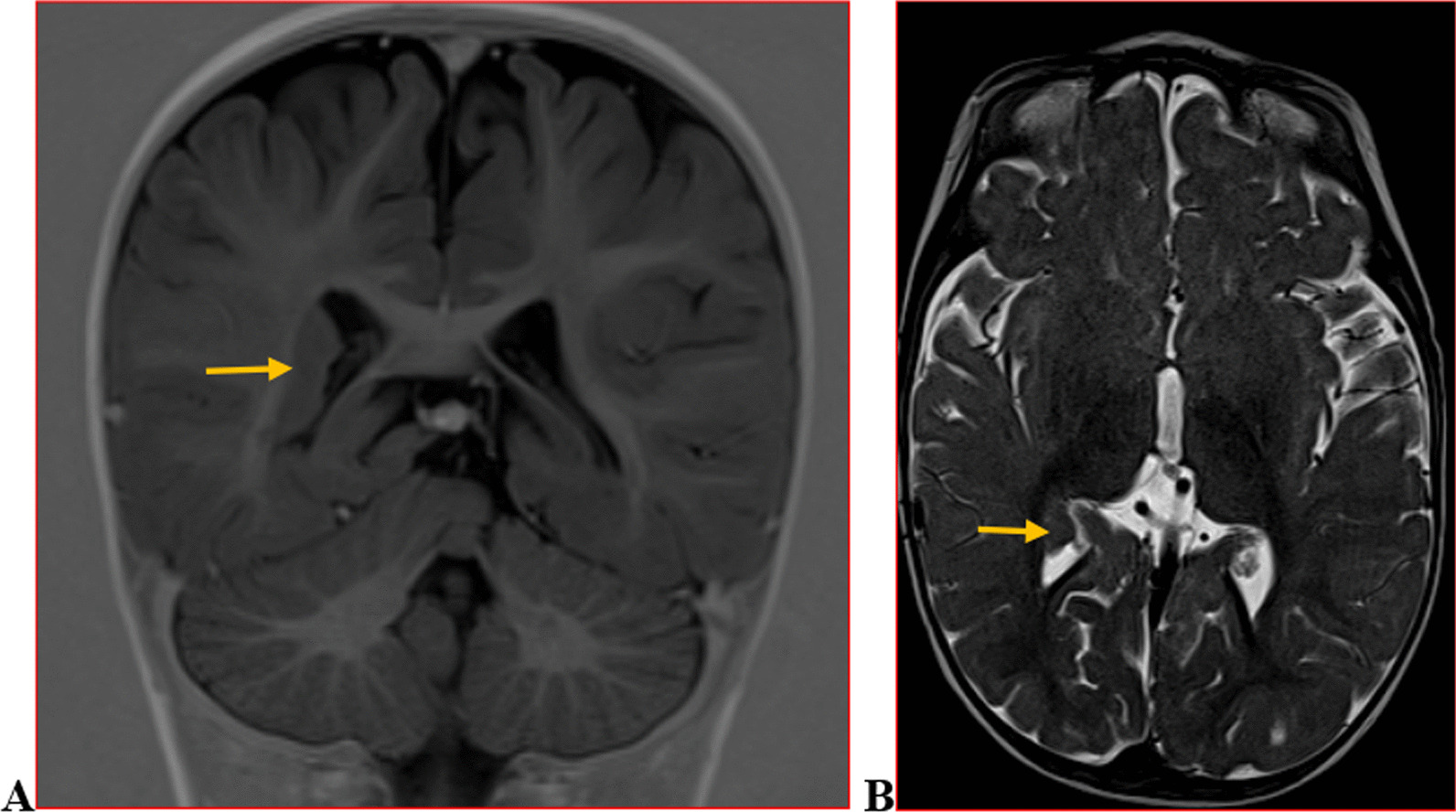
Subependymal heterotopia in an 18 month old male with focal seizures. Coronal T1 weighted (**A**) and axial T2 weighted (**B**) images demonstarting a lesion lining the right posterior horn and body of the lateral ventricle with grey matter signal intensity in all sequences (arrows)

**Fig. 13:**
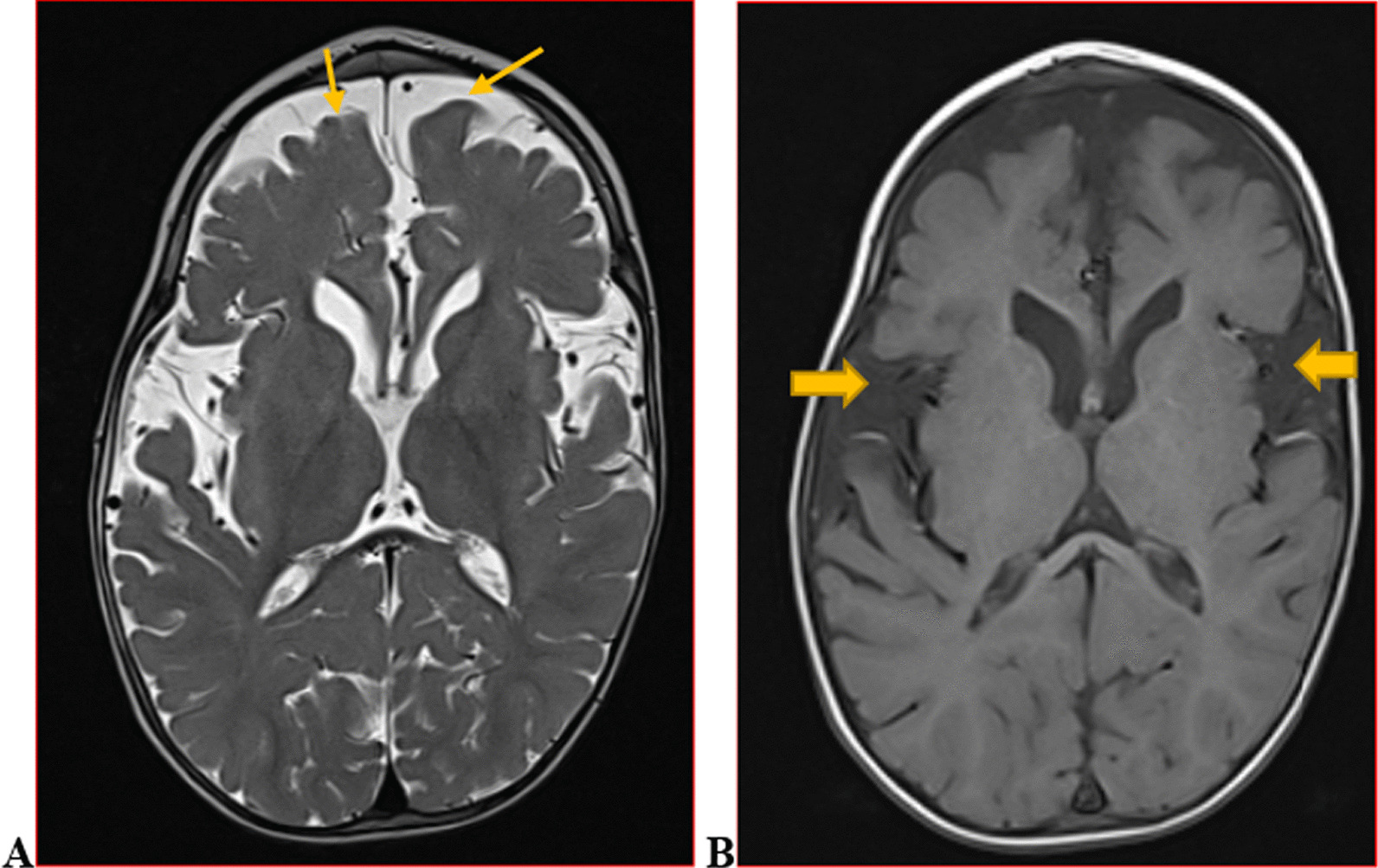
One and a half year old male with epilepsy and cranio-facial dysmorphism. Axial T2 (**A**) and T1 weighted (**B**) brain MRI showing delayed myelination with bilateral widened opercula and sylvian fissures with increased subarachnoid fluid (wide arrows).The frontal regions of the cerebral hemispheres are atrophied with enlarged anterior subarachnoid spaces(narrow arrows) in a case of type I glutaric aciduria

**Fig. 14:**
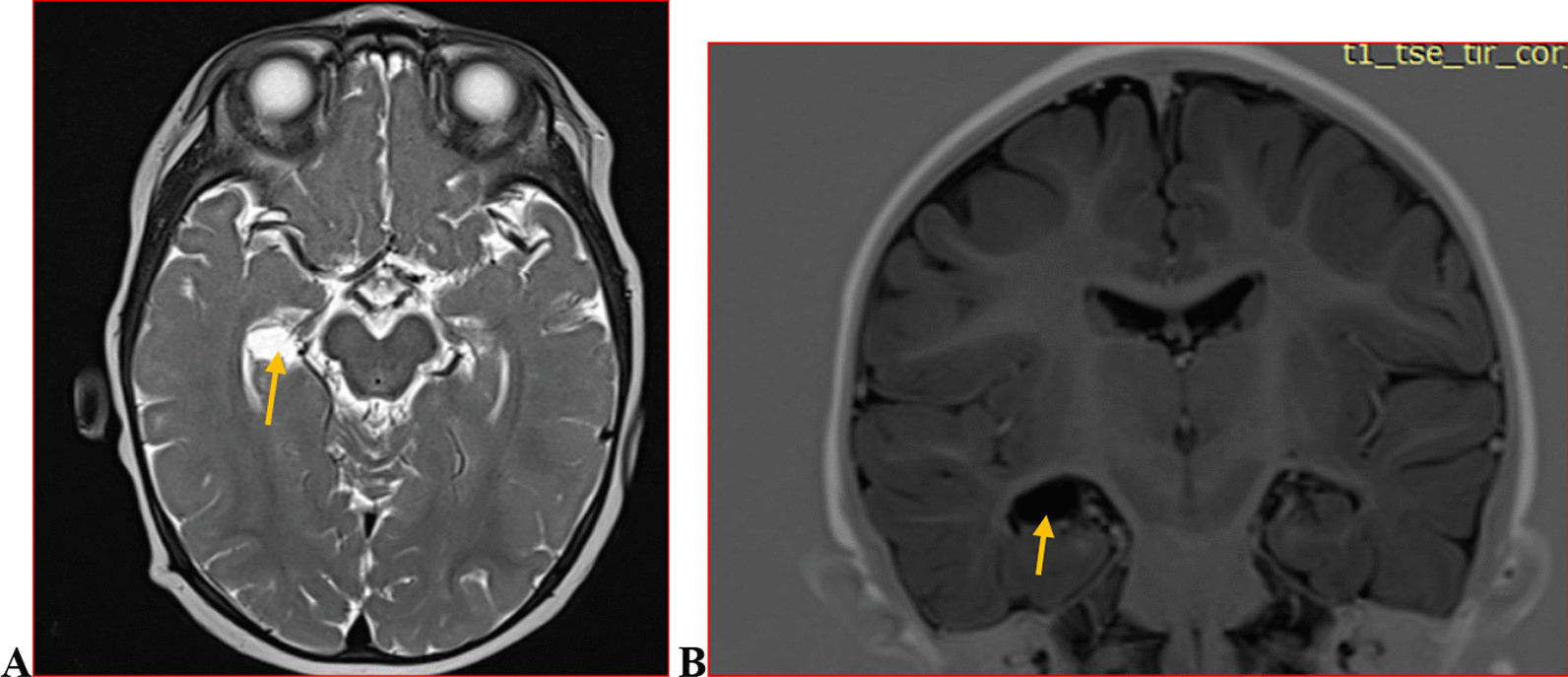
Axial T2 (**A**) and coronal T1 weighted (**B**) MRI demonstrating a well defined cyst with CSF signal intensity on all sequences in the plane of the right choroidal fissure (arrow) compressing the ipsilateral hippocampal head

**Fig. 15:**
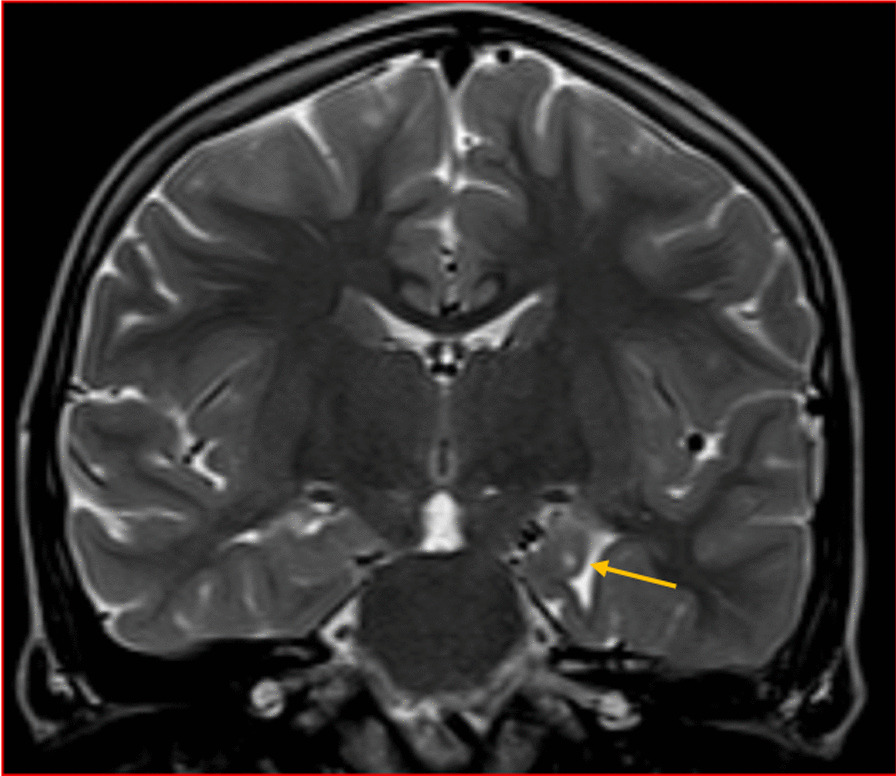
Six-year-old male with focal seizures. Coronal T2 weighted brain MRI demonstrating an abnormally shaped left hippocampal head (globular) with a vertically oriented collateral sulcus (arrow) in a case of incomplete left hippocampal inversion

**Fig. 16:**
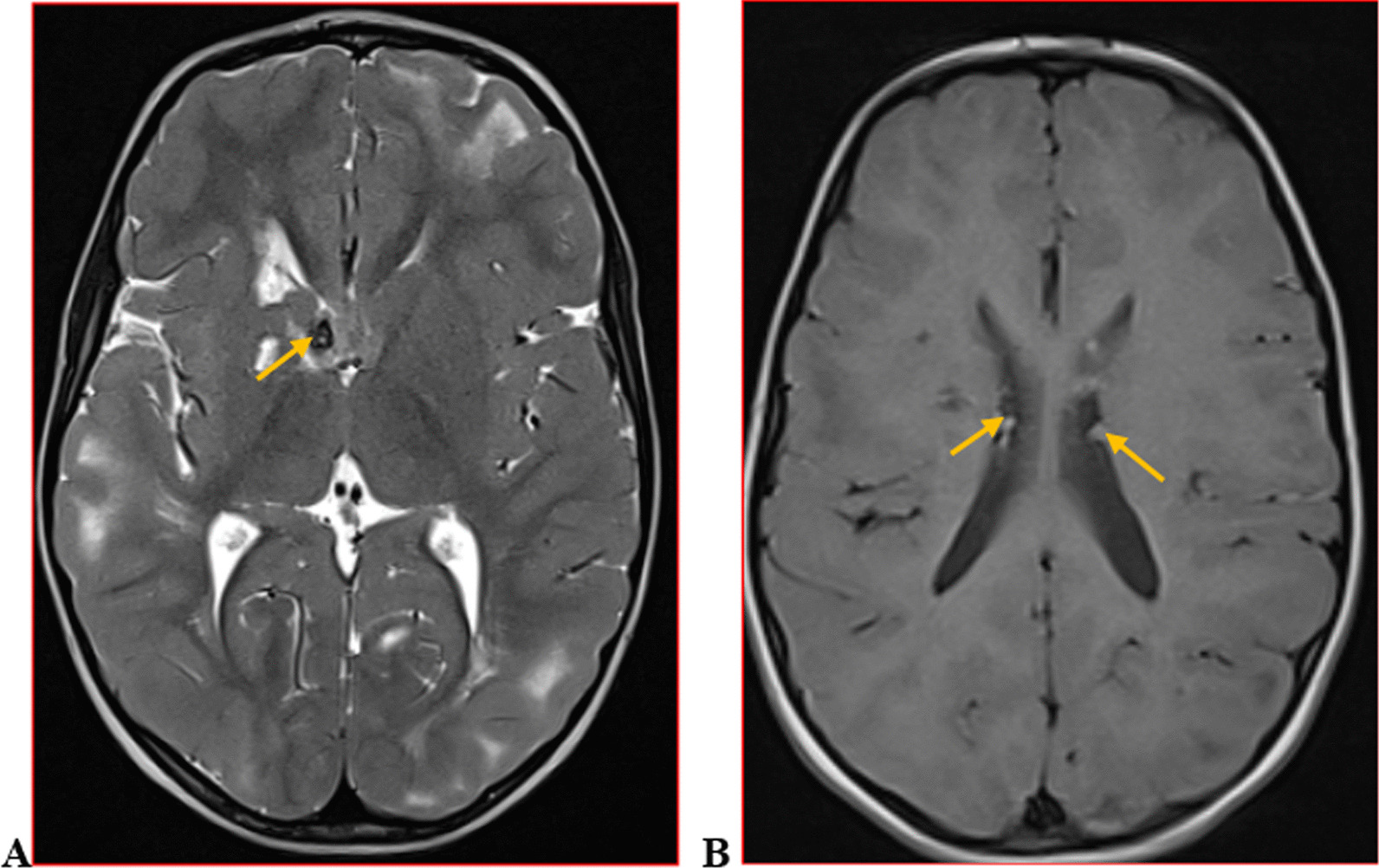
Tuberous Sclerosis Complex in a three year old male with epileptic spasms. Axial T2-weighted (**A**) brain MRI showing cortical/subcortical tubers as well-circumscribed areas of high signal intensity in the left frontal and occipital lobes and in the right temporal and occipital lobes representing subcortical tubers; T2-hypointense nodule in right foramen of Monroe representing a Subependymal Giant cell Astroytoma (SEGA) (arrow). Axial T1-weighted MRI (**B**) showing isointense nodules arising from the walls of the lateral ventricles (arrows) representing subependymal harmatomas

**Fig. 17:**
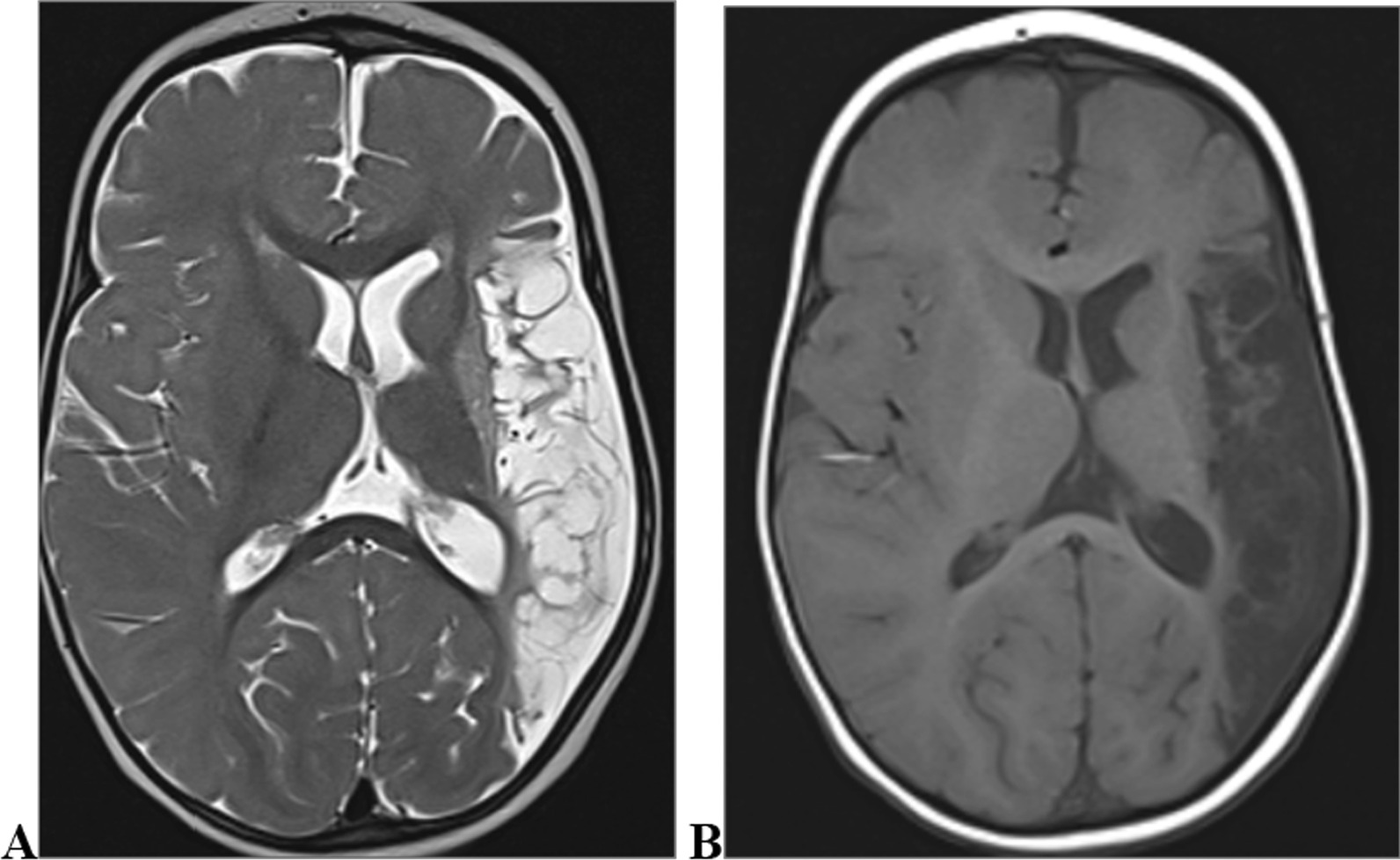
T2 weighted (**A**) and T1 weighted (**B**) axial images demonstrating left hemiatrophy with cystic encephalomalacia in the left middle cerebral artery territory as sequelae of neonatal stroke in a nine-month-old with right hemiplegia and seizures

We categorised the brain MRI abnormalities as congenital or acquired. Majority of structural abnormalities were acquired (61.22% of all abnormalities) as shown in Fig. 1. There were several acquired abnormalities detected in our study as seen in Table 1. The most common acquired abnormality was hippocampal sclerosis (HS) demonstrated in Fig. 2. Of the forty-seven participants with HS, there were 23 participants with left sided HS (Fig. 3), 22 with right sided HS and 2 with bilateral HS. Others included Hypoxic Ischaemic Encephalopathy (HIE) in Fig. 4 and Periventricular Leukomalacia (PVL) in Figs. 5, 6, 7, 8, 9, 10) neoplasm (Fig. 11), Kernicterus demonstrated in Fig. 6 and neonatal stroke ((Figs. 12, 13, 14, 15, 16, 17).

**Fig. 18:**
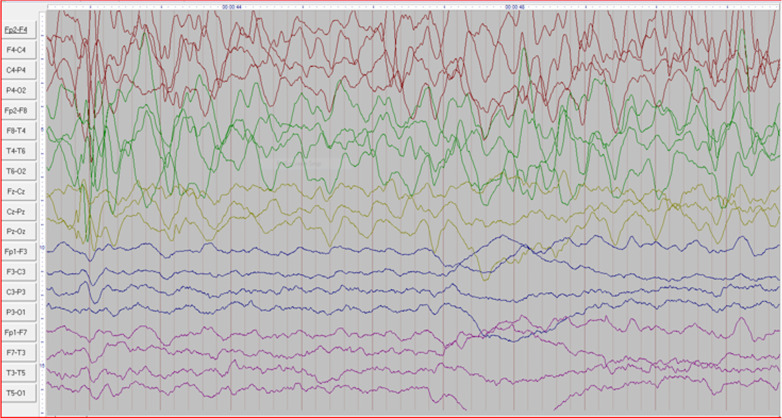
EEG corresponding to image above in the same child with neonatal stroke showing voltage asymmetry with significant slowing on the left side and sharp waves on the contralateral side

**Table 1:** Acquired MRI abnormalities in children with epilepsy who had brain MRI studies done at two urban hospitals in Kampala, Uganda

Acquired abnormalities	N (percentage)
Hippocampal sclerosis	48 (32.66%)
White matter abnormalities	2 (1.36%)
Acquired Hydrocephalus	2 (1.36%)
Infarction	8 (5.44%)
Anoxic brain injury	19 (12.92%)
Periventricular Leukomalacia(PVL)	16 (10.88%)
Hypoxic Ischemic Encephalopathy(HIE)	3 (2.04%)
Infection	2 (1.36%)
Meningitis	1 (0.68%)
Viral encephalitis*	1 (0.68%)
Autoimmune	
Rasmussen encephalitis	1 (0.68%)
Atrophy	3 (2.04%)
Neuro-metabolic disorders	7 (4.76%)
Kernicterus	6 (4.08%)
Hypoglycemia	1 (0.68%)
Neoplasms	4 (2.72%)

^*^Diagnosis of viral encephalitis was both clinical, laboratorial and with MRI

Among the congenital causes, disorders of cortical development were the most frequent. These included disorders of neurogenesis (microcephaly), disorders of migration (heterotropia seen in Fig. 12, lissencephaly seen in Fig. 7 and pachygyria) and disorders of organisation (hemimegalencephaly and focal cortical dysplasia (Fig. 3 and Fig. 9) as shown in Table 2. Others include aqueductal stenosis (Fig. 8), choroidal fissure cyst (Fig. 14), incomplete hippocampal inversion (Fig. 15) and Tuberous sclerosis complex (Fig. 16), neuro-metabolic disorders like Leigh disease (Fig. 10) and type I glutaric aciduria (Fig. 13).

**Table 2:** Congenital MRI abnormalities in participants with epilepsy who had brain MRI studies done at two urban hospitals in Kampala, Uganda

Congenital abnormalities	N(percentage)
1. Hippocampal abnormalities	
Incomplete Hippocampal inversion	1 (0.68%)
Choroidal fissure cyst	3 (2.04%)
Hippocampal sulcal remnant cyst	1 (0.68%)
2. Microcephaly*	1 (0.68%)
3. Disorders of cortical development	
Focal Cortical dysplasia	5 (3.4%)
Type 1	2 (1.36%)
Type 2	2 (1.36%)
Type 3	1 (0.68%)
Hemimegalencephaly	2 (1.36%)
Heterotopias	3 (2.04%)
Lissencephaly-pachygyria spectrum	5 (3.4%)
4. Neuro-metabolic disorders	3 (2.04%)
Leigh disease	1 (0.68%)
Unspecified	2 (1.36%)
5. Posterior fossa anomalies	3 (2.04%)
6. Aqueductal stenosis	5 (3.4%)
7. Corpus callosal dysgenesis	4 (2.72%)
8. Arachnoid cyst	1 (0.68%)
9. Tuberous sclerosis complex	2 (1.36%)
10. Holoprosenecephaly	1 (0.68%)
11. Colloid cyst	1 (0.68%)

^*^Microcephaly was clinically diagnosed and had radiological abnormalities

### EEG findings

83 out of 147 participants (56.46%) had an electroencephalogram (EEG) study done. Of these, 76 participants (91.6%) had abnormal findings and the rest had a normal EEG study.

The EEG abnormalities were grouped according to the source of discharge to include generalised17 (20.5%), focal45 (54.2%) and multifocal abnormalities14 (16.9%). We further classified the focal discharges according to the anatomical lobe source as shown in Table 3. Figure 18 demonstrates EEG findings in child with neonatal stroke.

**Table 3:** Bivariate analysis of demographic factors, electroencephalographic (EEG) findings and clinical history association with abnormal brain MRI findings

Variable	Total N (%)	Abnormal MRI	OR	95%CI	*p*
Age					
Infant	14 (9.52)	12 (11.01)	1.00		
Child 1-4 years	59 (40.14)	45 (41.28)	0.54	0.11–2.68	0.448
Child 5–9 years	47 (31.97)	36 (33.03)	0.54	0.11–2.81	0.469
Child 10–17 years	27 (18.37)	16 (14.68)	0.24	0.05–1.30	0.099
Sex					
Female	46 (31.29)	34 (31.19)	1.00		
Male	101 (68.71)	75 (68.81)	1.02	0.46–2.25	0.965
Region of descent					
Central Uganda	66 (44.90)	48 (44.04)	1.00		
Northern Uganda	11 (7.48)	10 (9.17)	3.75	0.45–31.42	0.223
Southern Uganda	5 (3.40)	4 (3.67)	1.50	0.16–14.34	0.725
Eastern Uganda	16 (10.88)	12 (11.01)	1.13	0.32–3.94	0.854
Western Uganda	38 (25.85)	26 (23.85)	0.81	0.34–1.94	0.641
Non-Ugandan	11 (7.48)	9 (8.26)	1.69	0.33–8.57	0.528
Birth asphyxia/perinatal trauma					
No	128 (87.07)	92 (84.40)	1.00		
Yes	18 (12.24)	16 (14.48)	0.32	0.70–1.46	0.141
EEG done					
No	64 (43.54)	49 (44.95)			
Yes	83 (56.46)	60 (55.05)	1.22	0.58–2.60	0.595
EEG abnormalities					
No	8 (9.64)	3 (5.00)	1.00		
Yes	75 (90.36)	57 (95.00)	5.28	1.15–24.28	0.033
EEG abnormalities distribution					
Generalised	18 (24.00)	13 (22.81)	1.00		
Focal	57 (76.00)	44 (77.19)	1.30	0.39–4.33	0.667
Frontal EEG abnormalities					
No	21 (36.84)	14 (31.82)	1.00		
Yes	36 (63.16)	30 (68.18)	2.50	0.71–8.83	0.155
Parietal EEG abnormalities					
No	29 (50.88)	22 (50.00)	1.00		
Yes	28 (49.12)	22 (50.00)	1.17	0.34–4.03	0.808
Occipital EEG abnormalities					
No	27 (47.37)	19 (43.18)	1.00		
Yes	30 (52.63)	25 (56.82)	2.11	0.59–7.47	0.249
Temporal EEG abnormalities					
No	12 (21.05)	10 (22.73)	1.00		
Yes	45 (78.95)	34 (77.27)	0.62	0.12–3.26	0.571

### Relationship between clinical, EEG and MRI findings

The largest number of children with epilepsy were in the one to four-year age group and they had the highest yield of structural brain abnormalities from the MRI study as shown in Table 3.

On bivariate logistic regression, EEG abnormalities was associated with abnormal brain MRI finding (see Table 3 for details).

Clinical factors of birth asphyxia/perinatal trauma 18 (12.24%), trauma 2 (1.36%) and history of CNS infection 2 (1.36%) were not significantly associated with abnormal brain MRI findings.

## Discussion

A prevalence of 74% of brain abnormalities among children with epilepsy found in our study is high in comparison to other studies within Africa and the developed world [18]. Various prevalence rates of 12–53% have been reported (ref). A similar study in Kenya reported a prevalence of 33% [19]. The high yield in our study could have been attributed to prior electroeencephalogram (EEG) studies which could have created a selection bias for the MRI studies. EEG is considered the primary means of preoperative determination of a seizure focus [20]. In addition, our study was done at tertiary hospitals with dedicated paediatric neurology clinics run by paediatric neurologists. Their selection of children for MRI studies tended to be those for whom the yield of abnormalities was likely to be high as opposed to other health workers.

Our study revealed a higher incidence of epilepsy in males as opposed to their female counterparts. This finding is similar to a report by Awa Ba-Diop et al. [21]. The reason for this is not clearly known. However, the possibility of genetics being at play is not excluded, and may warrant further inquiry. An epidemiology study done in Sub-Saharan Africa further alluded to the same with the exception of Tanzania where females outnumbered the males [9].

Majority of children with epilepsy were under five years. This finding is similar to previous reports by Awa Ba-Diop et al. and Ngugi et al. [9, 21]. The reason for this could be the increased susceptibility of the immature brain to seizures owing to the net effect of altered excitation-inhibitory balance in favour of enhanced excitation.

Acquired structural brain abnormalities (61.22%) were the most commonly seen lesion in our study. This findings is in keeping with previous Sub Saharan Africa studies by Awa Ba-Diop et al. and Ngugi et al. that reported factors such as birth asphyxia, traumatic brain injuries, malnutrition and neurological infection as the most common risk for epilepsy [9, 21]. In our study, radiological evidence of CNS infection was observed in two participants. This is a paradigm shift from previous reports and could be attributed to a couple of factors. Firstly, there could have been a selection bias of our study participants, since the poor might not have been able to afford the MRI study coupled with poor health seeking behaviour. Secondly, children with obvious structural abnormalities like HIE and hypoglycaemia are rarely imaged because of the preconceived cause. This could therefore have under played the burden of some epileptogenic structural abnormalities common to low resource settings including HIE and infections.

Hippocampal sclerosis (HS) was the commonest structural abnormality identified in our study. Varying rates of 8% to 30% have been reported [22–25]. The reason for this is not clearly understood. However, our study was performed with a dedicated epilepsy protocol which has been found to increase the detection of HS [26]. Secondly, reference to the EEG studies in our case could have provided a focus hypothesis directing the radiologist to look for abnormality in the hippocampal regions. This is in tandem with a study by Wehner et al. which found that correct diagnosis of HS on brain MRI studies was facilitated by knowledge of appearance of the lesion and prior provision of a focal hypothesis for the cause of the epilepsy [27].

Malformations of cortical development (MCD) were the most frequent congenital structural abnomalities at 8.84% with focal cortical dysplasias and lissencephaly-pachygyria spectrum having the highest number of three each. MCD are known to cause epilepsy as well as other complications such as global developmental delay [28]. Our findings were comparable to other studies [19]. In Kenya, a prevalence of 3.8% MCD was reported among children with epilepsy [19]. Focal cortical dysplasias were characterised by thickened cortical gyri with abnormal underlying white matter changes and blurred grey/white matter junction. This finding is usually subtle and a high index of suspicion is relevant for identification. Lissencephaly pachygyria spectrum is easier to identify as the brain is characteristically smooth with minimal to no gyration. Knowledge of the appearance of these lesions is of utmost importance for their correct diagnosis.

Four participants had brain neoplasms (2.72% of all abnormalities). This finding is similar to other studies which reported neoplasms as an etiology of epilepsy in children. Brain neoplasms account for 4–5% of all cases with epilepsy [29]. Liigant et al. (2001) reported a high seizure occurrence in children with brain neoplasms. Neoplasms should therefore be included in the differential for children presenting with seizures. Liigant et al. also found that the site of the tumour was associated with increased likelihood of seizure occurrence i.e. frontal and frontoparietal, temporal and frontotemporal, as well as the parietal and parasagittal regions. Our study findings were comparable, with the brain tumours located in the frontal and parietal lobes and one located in the midline.

Mitochondrial cytopathies is one of the recognised causes of epilepsy with structural brain abnormalities detectable on MRI. The commonest specific findings are of deep grey matter symmetrical T2/FLAIR hyperintensities and corresponding T1-hypointensity with diffusion restriction seen in acute lesions. In our study, ten participants were found to have brain MRI findings highly suggestive of mitochondrial/neurometabolic disease [30]. Patients may have accompanying structural changes such as hippocampal abnormalities seen in cases of chronic Kernicterus [31] as depicted in Fig. 6.

Electroencephalography (EEG) helps to detect interictal activity and localise the region of interictal activity and/or ictal activity [20, 32]. In our study,57.05% of the participants had an EEG study done. Of these, 55% had abnormalities detected on their brain MRI studies. We found that 95% of participants with an abnormal EEG also had epileptogenic structural abnormalities detected in their brain MRI studies. Dirik et al. reported 39.2% of participants in their study with abnormal EEG and MRI [33]. A study by Noh et al. found MRI abnormalities in 74% of participants with focal slowing and interictal epileptiform discharges [34]. The higher findings in our study compared to others could be due methodological differences.

### Limitations

Whilst our study is one of the first studies in Uganda to describe paediatric neuroimaging findings in children with epilepsy with a good sample size, we acknowledge some potential limitations. Our study was done at relatively good “tertiary” centres which run specialised paediatric neurology clinics with a possibility of a selection bias due to the financial hardships. This could affect the generalisability of the study findings.

## Conclusion

This study reaffirms the fundamental role of neuroimaging brain MRI studies in identification of structural causes of epilepsy. Furthermore, it shows that interictal EEG plays a role in raising the possibility of structural brain abnormality as the cause of epilepsy.

EEG can be used to complement decision making on priority for MRI imaging, as MRI is a scarce and costly resource in our setting.

The lesion localisation achieved by the combination of EEG and MRI will go a long way in pursuing epilepsy surgery as an option in the non-pharmacological treatment of medically intractable epilepsy.

## Data Availability

All data generated and/or analyzed during this study are available from the corresponding author on reasonable request.

## Abbreviations

MRI: Magnetic resonance imaging
MTS: Mesial temporal sclerosis
EEG: Electroencephalogram

## References

[1] Fisher RS, van Emde BW, Blume W, Elger C, Genton P, Lee P (2005). Epileptic seizures and epilepsy: definitions proposed by the International League Against Epilepsy (ILAE) and the International Bureau for Epilepsy (IBE). Epilepsia.

[2] Ngugi AK, Bottomley C, Kleinschmidt I, Sander JW, Newton CR (2010). Estimation of the burden of active and life-time epilepsy: a meta-analytic approach. Epilepsia.

[3] Newton CR, Garcia HH (2012). Epilepsy in poor regions of the world. Lancet.

[4] Scheffer IE, Berkovic S, Capovilla G, Connolly MB, French J, Guilhoto L (2017). ILAE classification of the epilepsies: position paper of the ILAE Commission for Classification and Terminology. Epilepsia.

[5] Recommendations for Neuroimaging of Patients with Epilepsy (1997). Commission on Neuroimaging of the International League Against Epilepsy. Epilepsia.

[6] Wilmshurst JM, Cross JH, Newton C, Kakooza AM, Wammanda RD, Mallewa M (2013). Children with epilepsy in Africa: recommendations from the International Child Neurology Association/African Child Neurology Association Workshop. J Child Neurol.

[7] Ackermann S, Le Roux S, Wilmshurst JM (2019). Epidemiology of children with epilepsy at a tertiary referral centre in South Africa. Seizure.

[8] Whelan CD, Altmann A, Botía JA, Jahanshad N, Hibar DP, Absil J (2018). Structural brain abnormalities in the common epilepsies assessed in a worldwide ENIGMA study. Brain.

[9] Ngugi AK, Bottomley C, Kleinschmidt I, Wagner RG, Kakooza-Mwesige A, Ae-Ngibise K (2013). Prevalence of active convulsive epilepsy in sub-Saharan Africa and associated risk factors: cross-sectional and case-control studies. Lancet Neurol.

[10] Barkovich AJ, Dobyns WB, Guerrini R (2015). Malformations of cortical development and epilepsy. Cold Spring Harb Perspect Med.

[11] Desikan RS, Barkovich AJ (2016). Malformations of cortical development. Ann Neurol.

[12] Tsai ML, Chen CL, Hsieh KL, Miser JS, Chang H, Liu YL (2018). Seizure characteristics are related to tumor pathology in children with brain tumors. Epilepsy Res.

[13] Daumas-Duport C (1993). Dysembryoplastic neuroepithelial tumours. Brain Pathol.

[14] Goncalves VT, Reis F, Queiroz Lde S, Franca M (2013). Pleomorphic xanthoastrocytoma: magnetic resonance imaging findings in a series of cases with histopathological confirmation. Arq Neuropsiquiatr.

[15] Gilliam F, Wyllie E (1996). Diagnostic testing of seizure disorders. Neurol Clin.

[16] Schramm J (2017). Seizures associated with cerebral arteriovenous malformations. Handb Clin Neurol.

[17] Bulut HT, Sarica MA, Baykan AH (2014). The value of susceptibility weighted magnetic resonance imaging in evaluation of patients with familial cerebral cavernous angioma. Int J Clin Exp Med.

[18] Berg AT. Epidemiology of seizure disorders. 2013.

[19] Samia P, Odero N, Njoroge M, Ochieng S, Mavuti J, Waa S (2021). Magnetic resonance imaging findings in childhood epilepsy at a Tertiary Hospital in Kenya. Front Neurol.

[20] Rosenow F, Klein KM, Hamer HM (2015). Non-invasive EEG evaluation in epilepsy diagnosis. Expert Rev Neurother.

[21] Ba-Diop A, Marin B, Druet-Cabanac M, Ngoungou EB, Newton CR, Preux PM (2014). Epidemiology, causes, and treatment of epilepsy in sub-Saharan Africa. Lancet Neurol.

[22] Wieshmann UC (2003). Clinical application of neuroimaging in epilepsy. J Neurol Neurosurg Psychiatry.

[23] Van Paesschen W, Sisodiya S, Connelly A, Duncan JS, Free SL, Raymond AA (1995). Quantitative hippocampal MRI and intractable temporal lobe epilepsy. Neurology.

[24] Hakami T, McIntosh A, Todaro M, Lui E, Yerra R, Tan KM (2013). MRI-identified pathology in adults with new-onset seizures. Neurology.

[25] Liu RS, Lemieux L, Bell GS, Sisodiya SM, Bartlett PA, Shorvon SD (2002). The structural consequences of newly diagnosed seizures. Ann Neurol.

[26] Ponnatapura J, Vemanna S, Ballal S, Singla A (2018). Utility of magnetic resonance imaging brain epilepsy protocol in new-onset seizures: how is it different in developing countries?. J Clin Imaging Sci.

[27] Wehner T, Weckesser P, Schulz S, Kowoll A, Fischer S, Bosch J (2021). Factors influencing the detection of treatable epileptogenic lesions on MRI. A randomized prospective study. Neurol Res Pract..

[28] Fernández-Menéndez A, Casado A (2016). Review and update on malformations of cortical development and neuronal migration disorders. Pediatr Adolescent Med.

[29] Hauser WA, Annegers JF, Kurland LT (1993). Incidence of epilepsy and unprovoked seizures in Rochester, Minnesota: 1935–1984. Epilepsia.

[30] Pereira FV, Jarry VM, Castro JTS, Appenzeller S, Reis F (2021). Pediatric inflammatory demyelinating disorders and mimickers: How to differentiate with MRI?. Autoimmun Rev.

[31] Bisinotto HS, Jarry VM, Reis F (2021). Clinical and radiological aspects of bilateral temporal abnormalities: pictorial essay. Radiol Bras.

[32] Flink R, Pedersen B, Guekht AB, Malmgren K, Michelucci R, Neville B (2002). Guidelines for the use of EEG methodology in the diagnosis of epilepsy. International League Against Epilepsy: commission report. Commission on European Affairs: Subcommission on European Guidelines. Acta Neurol Scand.

[33] Dirik MA, Sanlidag B (2018). Magnetic resonance imaging and interictal electroencephalography findings in newly diagnosed epileptic children. J Clin Med.

[34] Noh BH, Berg AT, Nordli DR (2013). Concordance of MRI lesions and EEG focal slowing in children with nonsyndromic epilepsy. Epilepsia.

